# Editorial: Polymeric Nano-Biomaterials for Medical Applications: Advancements in Developing and Implementation Considering Safety-by-Design Concepts

**DOI:** 10.3389/fbioe.2020.599950

**Published:** 2020-10-19

**Authors:** Gerrit Borchard, Claudia Som, Manfred Zinn, Vasile Ostafe, Olga Borges, Giuseppe Perale, Peter Wick

**Affiliations:** ^1^School of Pharmaceutical Sciences, Université de Genève, Geneva, Switzerland; ^2^Swiss Federal Laboratories for Materials Science and Technology, Dübendorf, Switzerland; ^3^Institute of Life Technologies, University of Applied Sciences and Arts Western Switzerland (HES-SO Valais-Wallis), Sion, Switzerland; ^4^Advanced Environmental Research Laboratories, Department of Biology-Chemistry, Faculty of Chemistry, Biology, Geography, West University of Timisoara, Timisoara, Romania; ^5^Centre for Neuroscience and Cell Biology, University of Coimbra, Coimbra, Portugal; ^6^Department of Innovative Technologies, University of Applied Sciences and Arts of Southern Switzerland (SUPSI), Manno, Switzerland

**Keywords:** nano-biomaterials, safe-by-design, nanotechnology, nanomedicines, drug carriers

The aging population represents an enormous social, structural, and financial burden on society. To address the potential problems of supporting an elderly population, it is important to consider ways of enabling individuals to maintain independence and quality-of-life (QoL) for as long as possible. To achieve this, we need to develop new solutions and novel concepts through technologies, and researchers have been exploring how disease and aging are mediated by molecular processes at the nanoscale. The subsequent nanotechnology-enabled approaches that have emerged in recent years include innovative nano-materials and nano-devices, which could enable interventions at the molecular length scale and are, therefore, an important cornerstone in building solutions.

As for every novel technology or material, a careful safety assessment is needed early in its development to avoid social and economic drawbacks. While guidelines and legislation have been put in place for therapeutics of low molecular weight along with much more complex biologics as well as their follow-on products (generics and biosimilars, respectively), the regulatory approach for advanced therapeutics including polymeric biomaterials is still in its infancy. The rise of so-called “nanomedicines” or complex therapeutics along with follow-on products such as “nanosimilars” or “complex generics” have provided advantageous attributes on the nanoscale, but the lack of guidance is a difficult challenge that needs to be addressed in the development of such products in future.

This issue describes some of the different nano-biomaterials that are currently being studied for the preparation of nanoscale drug carriers. These include: chitosan (see Jesus, Marques et al.; Marques et al.), a chitin derivative biopolymer obtained mostly from crustaceans; the family of poly(lactide-*co*-glycolide) (PLGA) polymers (Essa et al.); poly(D,L-lactic acid) (Casalini, Rossi et al.; Da Silva et al.); polyhydroxyalkanoates (Casalini, Rosolen et al.), a polyester produced by bacteria; and hydrogel systems made up of hyaluronic acid and alginate (Lynch et al.). As indicated in this issue, some studies have found that for some materials [e.g., chitosan (Marques et al.)], the description of the specifications of the nano-biomaterial is often incomplete. The preparation of nano-vectors using these materials is also complex that needs to be better understood, in particular when self-assembly processes are used to implement therapeutic drug delivery (Yadav et al.). These issues render comparative efficacy studies difficult and preclude the introduction of a “safe-by-design” concept, as developed in the framework of the European project “GoNanoBioMat” (https://gonanobiomat.eu/). As a concept, safe-by-design is currently being adapted for use in research on nano-biomaterials, based on the processes used in drug discovery and development, which adopt safety aspects early in the process (Schmutz et al.). It is intended that this concept in combination with other existing regulatory frameworks, will guide small and medium-sized companies during the development process of nanomedicines, span stages from material selection and design, characterization, assessment of human, and environmental health risks, to manufacturing and control, as well as storage and transport of the final product.

This special issue also focuses on the hazard assessment of polymeric biomaterials for medical use [see the literature study (Jesus, Schmutz et al.)], and the determination of the impact of certain properties of nanoscale drug vectors on their safety (cytotoxicity, immunotoxicity) (Jesus, Marques et al.), which discuss membrane diffusion characteristics and pharmacokinetics. Some of these parameters, including nanomaterial interactions with their biological environment (Casalini, Rosolen et al.) and the evaluation of the risks of their degradation products (Roman et al.) can be assessed by computational simulation (Casalini, Limongelli et al.) or systematic evaluation of animal studies (Hauser and Nowack), resulting in concrete suggestions for nano-biomaterial design to achieve optimized efficacy and enhanced safety. Finally, the different options of targeted anti-schistosomal therapy using nanotechnology are reviewed (Adekiya et al.).

The diversity of the different papers presented in this special issue are indicative of the significant interplay between life and the material sciences with computational approaches. We believe that this exchange of ideas is one of the best approaches to tackling the increasingly complex challenges of an aging society.

## Author Contributions

GB has drafted the manuscript and submitted in final form. CS, MZ, VO, OB, GP, and PW have revised the manuscript draft. All authors contributed to the article and approved the submitted version.

## Conflict of Interest

The authors declare that the research was conducted in the absence of any commercial or financial relationships that could be construed as a potential conflict of interest.

